# Topical clobetasol for the treatment of toxic epidermal necrolysis: study protocol for a randomized controlled trial

**DOI:** 10.1186/s13063-015-0879-7

**Published:** 2015-08-22

**Authors:** Reason Wilken, Chin Shang Li, Victoria R. Sharon, Kyoungmi Kim, Falin B. Patel, Forum Patel, Emanual Maverakis

**Affiliations:** Department of Dermatology, University of California, Davis School of Medicine, 3301 C Street, Suite 1400, Sacramento, CA 95816 USA

**Keywords:** Apoptosis, Clobetasol, Stevens–Johnson syndrome, Topical steroids, Toxic epidermal necrolysis

## Abstract

**Background:**

Toxic epidermal necrolysis (TEN) is a rare systemic allergic drug eruption with high patient mortality. Currently, no established treatments have been shown to be effective for TEN beyond supportive care. Prior studies of systemic corticosteroids have yielded conflicting data, with some showing a possible benefit and others reporting in increased mortality. However, topical steroids have shown promise for treatment of ocular sequelae of TEN, such as scarring and vision loss. We have designed a randomized controlled trial to evaluate topical clobetasol for treatment of the epidermal manifestations of TEN. In addition, we propose genetic studies to characterize the TEN transcriptome and alterations in cutaneous gene expression that might occur following topical steroid treatment.

**Methods/Design:**

This split-body randomized, double-blind, placebo-controlled Phase IIa proof-of-concept trial will evaluate the safety and efficacy of once-daily topical clobetasol applied to the skin of patients with TEN. This multicenter trial will recruit a total of 15 patients between the ages of 12 and 85 from the University of California Davis Medical Center and Shriners Hospital for Children inpatient burn units. Designated treatment areas on opposite sides of the body will be treated with blinded clobetasol 0.05 % ointment or control petrolatum ointment daily for 14 days. On day 3 of therapy, a biopsy will be taken from the treated area for genetic studies. The primary study aims will be to establish the safety of topical clobetasol treatment and determine the time to cessation of skin detachment for the control and clobetasol-treated areas. Secondary endpoints will evaluate efficacy using parameters such as time to 90 % re-epithelialization and percentage of affected skin at 0, 3, 6, 9, 12 and 15 days. Genomic DNA and RNA will be obtained from biopsy samples, to characterize the TEN transcriptome and identify changes in gene expression after topical steroid treatment.

**Discussion:**

Topical steroids have shown promise for treating ocular complications of TEN, but to date have not been evaluated for cutaneous manifestations of the disease. This trial will investigate clinical and molecular outcomes of topical clobetasol application and hopefully provide insight into the disease pathophysiology.

**Trial registration:**

ClinicalTrials.gov NCT02319616. https://clinicaltrials.gov/ct2/show/NCT02351037

## Background

Toxic epidermal necrolysis (TEN) is a rare systemic immune-mediated drug eruption predominantly affecting the skin, eyes, and genital and oral mucosa [1–4]. The pathogenesis of TEN occurs as a result of keratinocyte apoptosis leading to full-thickness epidermal necrosis and subsequent widespread epidermal detachment from the underlying dermis. Toxic epidermal necrolysis may be caused by a variety of medications, most commonly anti-epileptic agents, antibiotics, and allopurinol; however, non-steroidal anti-inflammatory drugs and various antihypertensive agents have also been implicated [5–8]. There is an annual incidence of 0.4 to 1.3 cases per million people worldwide [9–12]. Mortality is very high (20–50 %), owing to acute complications including sepsis, hypovolemic shock, and multisystem organ failure. Survivors of TEN might also suffer from long-term sequelae, such as permanent vision loss and adhesions of the genitalia [9, 13–16]. While some experts argue that Stevens–Johnson syndrome (SJS) and TEN represent distinct entities, others define SJS and TEN on a continuum differing only on the basis of involved body surface area, with SJS involving less than 10 % eroded body surface area, SJS–TEN overlap involving 10–30 % eroded body surface area and TEN involving greater than 30 % eroded body surface area [17–19].

Currently, there are no established therapies for SJS and TEN beyond supportive care. Multiple immunomodulatory and immunosuppressive agents have been used in clinical practice for the treatment of TEN, including systemic corticosteroids, intravenous immunoglobulin therapy, plasmapheresis, cyclosporine, and inhibitors of TNF including infliximab and etanercept [20–27]. With the exception of thalidomide (which was shown to be harmful), no therapies for TEN have been studied in randomized controlled trials [28]. The use of systemic corticosteroids for the treatment of TEN is controversial, with varying outcomes in published case studies and no randomized controlled trials to date [29]. Early studies of systemic corticosteroids for TEN showed an increase in complications and mortality rate relative to supportive care alone in burn care centers [30, 31]. Owing to the lack of evidence supporting systemic steroids for TEN and the risk of potential complications, such as sepsis and delayed re-epithelialization, they are not used in the current standard of care. However, glucocorticoids are known to inhibit keratinocyte apoptosis by inducing expression of anti-apoptotic genes and suppressing expression of pro-apoptotic genes, such as TNF and granulysin [32–34]. Given the pathologic mechanism of keratinocyte death in TEN, corticosteroids might hold important therapeutic potential.

Topical steroids have been evaluated for treatment of ocular complications of TEN including corneal ulceration, episcleritis, and conjunctivitis [16, 35]. Corticosteroids applied directly to the eyes have been shown in multiple studies to shorten duration and decrease the incidence of long-term sequelae, such as ocular cicatrization and vision loss [16, 35, 36]. The skin and eyes are affected by the same pathologic process in TEN; thus, topical steroids might also hold promise for treating the cutaneous manifestations of the disease. In addition, a topical delivery system might reduce the incidence of adverse events that have limited previous studies of systemic corticosteroids in TEN. The experimental focus of this trial is, therefore, to evaluate the safety and clinical outcomes of topical clobetasol applied to the skin of patients with TEN.

## Methods

### Design

The study is designed as a randomized, placebo-controlled, double-blind split-body Phase IIa proof-of-concept clinical trial to investigate the safety and efficacy of topical clobetasol 0.05 % ointment for the treatment of TEN. A total of 15 patients will be enrolled from the University of California Davis Burn Center or the Northern California Shriners Hospital for Children after being admitted with a diagnosis of biopsy-proven TEN. The initial diagnostic biopsy will be performed by the primary treating team prior to trial enrollment as per standard of care, to establish the diagnosis of TEN. The intervention will be once-daily application of either topical clobetasol 0.05 % ointment (‘treatment’) or inert petrolatum ointment (‘placebo’) to designated areas on opposite sides of the body for a total of 14 days or until the patient is discharged from the hospital. Treatment and placebo areas will each comprise 5 % or less of total body surface area and the locations will be designated based on areas of comparable disease severity. The selection of treatment areas on opposite sides of the body will minimize any local cross-contamination between clobetasol and placebo-treated sites. By virtue of the split-body design, patients will serve as their own controls. To characterize the TEN transcriptome, skin biopsies will be obtained from the clobetasol and placebo-treated areas on day 3 of treatment and used to obtain genomic DNA, RNA, and miRNA. The RNA samples will be used to generate sample libraries for Illumina sequencing, so the gene expression profile can be compared between the control and clobetasol-treated skin.

### Recruitment of patients

Patients will be recruited from the inpatient population at both the University of California Davis Burn Center and the Northern California Shriners Hospital for Children. As detailed in the inclusion criteria, patients must be admitted to either facility with a diagnosis of biopsy-proven TEN supported by a variety of additional clinical and pathologic findings (Table 1). We plan to enroll a total of 15 patients between the ages of 12 and 85 years in the trial. Review of the University of California Davis dermatology records reveals approximately 14 newly diagnosed cases of TEN per year (defined as body surface area > 10 %) between these two clinical sites, with roughly 10 patients per year meeting the inclusion criteria as defined in Table 2. Severely ill patients with greater than 70 % eroded skin or a SCORETEN (SCORE of Toxic Epidermal Necrosis, Table 2) greater than 3 (which is associated with >36 % mortality rate) at the time of hospital admission will not be eligible for enrollment into the study. We thus estimate the recruitment phase to last between 16 and 22 months. Owing to the severity of the disease and the history of poor trial outcomes for past TEN therapies [28], patients will be enrolled in two stages. Initially, five patients will be enrolled in the study. If three or more patients survive and at least three patients have improved time to cessation of skin detachment, then the additional ten patients will be enrolled.

**Table 1:** Study inclusion and exclusion criteria

Inclusion criteria	Exclusion criteria
Diagnosis of TEN by all of the following:	Patients younger than 12 or older than 85
• Characteristic histologic findings on diagnostic biopsy	Patients who have documented:
• Clinical diagnosis verified by two independent physicians	• Uncontrolled infection (for example, documented bacteremia)
• Greater than 10 % eroded body surface area	• Malignancy
• Negative pregnancy test in reproductive-age female patients	• Known prior immunodeficiency
• Actively worsening disease (enlarging area of involvement or new erosions occurring over the previous 24 hours)	• Pregnancy
• Patient body surface area > 1.0 m^2^	• Concurrent use of systemic corticosteroids in the burn center greater than or equal to 0.5 mg/(kg day) or prednisone or equivalent dose of systemic corticosteroid
	• Greater than 70 % eroded skin
	• Hepatitis
	• SCORETEN > 3 on admission
	• Active hepatitis, or alanine transaminase or aspartate aminotransferase above four times normal limits
	• Renal insufficiency (glomerular filtration rate < 50 ml/(min*1.73 m^2^))

**Table 2:** SCORETEN grading system for disease severity in TEN

Score	Criteria
1	Age > 40
1	Presence of a comorbid malignancy
1	Heart rate > 120 beats per minute
1	Initial percentage of epidermal detachment >10 %
1	Serum urea level >10 mmol/l
1	Serum glucose level >14 mmol/l
1	Serum bicarbonate level <20 mmol/l

### Ethical approval

The study protocol was reviewed and approved by the University of California Davis Institutional Review Board in December 2014 (#642415).

### Inclusion and exclusion criteria

Please refer to Table 1 for a listing of study inclusion and exclusion criteria.

### Informed consent process

Patients admitted to either University of California Davis Burn Center or Shriners Hospital for Children with biopsy-proven TEN will be asked if they would like to participate in the study. Patients will be informed that the participation in this study is completely voluntary and they may choose to withdraw at any time. They will also be informed that agreement or refusal of participation will not affect their care. If patients are interested, the study will be explained to them by the principal investigator or another University of California Davis physician listed on the Institutional Review Board and it will be determined whether the patient meets the study inclusion criteria. When eligible patients are interested in participating after a full discussion of risks and benefits, informed written consent will be obtained and stored in the their medical records.

Written consent for minors (<18 years of age) will be obtained from their parents or legal guardians. If no parent or designated legal guardian consent is available to discuss the study and provide consent, the minor will not be eligible for enrollment. In addition, in minors <18 years of age, assent of the minor will also be taken into account in determining the minor’s interest in participating in the study. For adult patients who are unable to provide consent for medical reasons (such as altered mental status, intubation or sedation), a family member or designated healthcare power of attorney may be able to provide consent on behalf of the patient. Patients who do not have available family members or individuals with legal authority to direct their healthcare will not be eligible for enrollment in the study. If otherwise eligible medically incapacitated individuals are enrolled in this manner via consent by family member or legal guardian, they will be informed of their enrollment in the study upon recovery of competent mental status and given the option to continue or withdraw from the study after full explanation of the potential risks and benefits.

### Study methods and interventions

#### Clinical studies

Enrolled patients will have an affected area of skin (comprising 5 % or less of total body surface area) on the right side of the body randomly assigned to receive either clobetasol 0.05 % ointment or control petrolatum ointment by means of computer randomization. A comparably involved treatment area on the left side of the body (also comprising 5 % or less of the total body surface area) will be designated to receive by default the unassigned treatment. The study pharmacist will conduct the randomization and therefore determine the treatment regimen for each area. The physicians and nurses will be blinded to the treatment. The dosing of clobetasol ointment in grams is listed in Table 3 and is based on the patient’s body surface area. The amount of clobetasol ointment to be applied is based on a French study describing topical steroid dosing for bullous pemphigoid [37]. In this study, subjects had a total of 40 g of clobetasol applied to the entire body twice daily, including the areas with eroded skin. The mean treatment duration in this study was 106 days. Body surface area was not specified in this study, which was conducted on elderly French women, but it is likely to be less than the average body surface area for an American patient with TEN.

**Table 3:** Dosing of clobetasol by body surface area

Body surface area (m^2^)	Clobetasol (g)
>1.8 m^2^	6
1.5–1.8 m^2^	4
1.0–1.5	3
<1.0	Will not be enrolled

Clobetasol is currently approved by the US Food and Drug Administration (FDA) for topical application up to twice daily, with a maximum dosage of 50 g per week. We have calculated the dosage of clobetasol to be applied based on total body surface area, and further adjusted the amount to fall within the maximum FDA-approved dosing regimen of 50 g per week. Based on the above regimen of 40 g twice daily for the entire body (and assuming an average body surface area of 1.5–1.8 m^2^ for the previous French study), we have calculated the clobetasol dose that should be applied to an area of 5 % body surface area as: 40 g × 0.05 (to adjust for 5 % body surface area) × 2 (to adjust for once-daily dosing) = 4 g daily for patients with a body surface area of 1.5–1.8 m^2^. Patients in the range of 1.0–1.5 m^2^ will receive an adjusted dose of 3 g daily, and patients in the highest body surface area category (>1.8 m^2^) will receive 6 g of clobetasol daily, in order to remain within the maximum dose of 50 g weekly.

The designated areas will be treated with the blinded clobetasol or petrolatum ointments daily for a total of 14 days or until discharge from the hospital. Following application of the ointments, the treated areas will be dressed with antibacterial xeroform dressings and sterile gauze. The burn unit attended will be allowed to prescribe any additional treatment that is medically indicated or part of their usual treatment regimen, such as occlusive dressings, antibacterial ointments, or skin xenografts. However, both of the designated treatment areas must receive the same supportive therapies in order for the patient to remain in the study. For example, a xenograft or a topical antibacterial ointment may be applied bilaterally if clinically indicated. However, if such additional treatment is prescribed for only one of the treatment areas, the patient will be withdrawn from the study.

Daily skin evaluation will be performed by trained study personnel prior to ointment application. Each treatment area will receive a numeric cellulitis score (Table 4) to allow for detailed monitoring and recognition of clinical signs of disease progression or development of local cellulitis. These values will be recorded at each daily skin evaluation before ointment application. The time to cessation of skin detachment (in days) is a primary endpoint measure of the study and will be determined through daily clinical examinations including assessment for the Nikolsky sign (separation of the superficial epidermis and blister formation resulting from direct manual pressure). As secondary measures of efficacy, the following variables will be recorded for each patient: time to 90 % re-epithelialization, percentage affected body surface area, and percentage surface area of detached skin. All secondary values for efficacy will be evaluated at 0, 3, 6, 9, 12, and 15 days. To our knowledge there are no validated outcome measures for TEN; however, prior clinical studies have used similar efficacy outcomes. Patients will also be photographed daily.

**Table 4:** Numeric cellulitis score

Solicited events or presence of:	Score	Assessment
Erythema	1	Pink or normal for ethnic group
	2	Bright red and/or blanches to touch
	3	White grey pallor or hypopigmented
	4	Dark red or purple and/or nonblanchable
	5	Black or hyperpigmented
Edema	1	None
	2	Minimal swelling
	3	Nonpitting edema
	4	Pitting edema
	5	Crepitus
Itchiness	1	None
	2	Noticeable
	3	Interrupted activities

Since there are multiple cutaneous diseases with similar presentations (such as erythema multiforme and staphylococcal scalded skin syndrome), TEN is usually diagnosed with the aid of a skin biopsy [38, 39]. Thus, the initial biopsy needed to confirm the diagnosis of TEN is part of the routine standard of care. If a patient has not yet had a diagnostic biopsy at the time of dermatology consultation, one will be performed to confirm the diagnosis and satisfy enrollment criteria. Patients enrolled in the study will have 4 mm punch biopsies from both treatment areas performed on day 3 to provide skin samples for molecular studies, as described next, to characterize the TEN transcriptome in control and steroid treated skin. The skin will be closed in standard fashion with non-absorbable nylon sutures that will be removed in 5–7 days.

#### Molecular studies

Biopsy specimens will be placed in RLT-plus buffer (Qiagen) with 1 % mercaptoethanol and Qiazol (Qiagen) and then powderized at −150 °C. Large RNA (>200 nucleotides), miRNA (<200 nucleotides) and genomic DNA will be extracted according to the manufacturer’s instructions (Qiagen). A volume of 2.5 μl of reaction buffer (Dilution Buffer 19: RNase inhibitor 1) from the SMARTer Ultra Low RNA Kit (Illumina Sequencing Clontech) will be added to the RNA solution, which will then be partially dried to a volume of 2.5–3.5 μl. Complementary DNA synthesis, purification, amplification, and shearing will be performed following the protocol included in the SMARTer Ultra Low RNA Kit for Illumina Sequencing. The Illumina HiSeq DNA sample preparation kit will be used for making the sample library. The DNA ends will be repaired; 3′ ends will be adenylated and then ligated to adaptor sequences. The DNA will then be purified and amplified by PCR. Complementary DNA sequences will be analyzed by the Illumina GAIIx system. ‘Bowtie’ will be used to align the enormous number of short DNA sequences. ‘TopHat’ will map DNA sequences using splicing junction information. Gene expression levels will be calculated using ‘Cufflinks.’ Bowtie, TopHat, and Cufflinks are distributed by the University of California Davis Center for Bioinformatics and Computational Biology. CASAVA can analyze SNP from whole transcriptome data. We will identify genes that are drastically changed in TEN and by the application of topical steroids. If we find informative SNPs on mRNA, we will analyze the corresponding site of genomic DNA. We will analyze miRNA sequences and the corresponding DNA sequencing of the miRNAs using the TruSeq Small RNA Sample Prep Kit and Illumina GAIIx system. Confirmation of interesting results from the sequencing experiment will be made with real-time PCR and immunohistochemistry.

### Primary endpoint measures

#### Time to cessation of skin detachment

This time will be determined through daily systematic skin examinations of the designated treatment areas and will be recorded in days.

#### Safety

A possible concern of this study is an increased rate of infection, both in the form of local cellulitis at the sites of clobetasol application or increased frequency of systemic infections based on expected rates for disease severity. Overall patient status will be evaluated and detailed skin examinations will be performed daily to generate a numeric cellulitis score (Table 4).

### Secondary endpoint measures

The secondary endpoint measures relate to the efficacy of topical clobetasol in reducing inflammation, shortening disease duration and promoting skin healing in patients with TEN.

#### Time to 90 % re-epithelialization

This time will be determined based on daily skin examinations and recorded in days.

#### Percent affected surface area

This will be recorded at 0, 3, 6, 9, 12 and 15 days.

#### Percent surface area detached skin

This will be recorded at 0, 3, 6, 9, 12 and 15 days.

### Reporting of adverse events

The data safety monitoring board assigned by the Clinical and Translational Science Center will review the cellulitis score (Table 3) data closely to assess for adverse events. The clinical presentation of TEN might make it difficult to distinguish disease worsening from local infection, as both processes can have a similar appearance. To distinguish local cellulitis versus global disease worsening, the treatment areas will be compared. If the total cellulitis score for one area is 30 % greater than the other area, it will be considered significant for possible cellulitis. If no external cause can be identified, such as a pressure-induced sore or infiltrated intravenous site, then the ointment applied to the affected area will be discontinued and this will be recorded as a safety outcome. Patients who require treatment discontinuation to either area will be followed longitudinally and will be evaluated on an ‘intention-to-treat’ basis.

Patients enrolled in the study will be closely monitored following the burn unit’s standard of care, including assessment for signs and symptoms of systemic infection. Patients in the burn unit routinely have blood cultures performed as part of this ongoing monitoring. We do not anticipate that topical clobetasol will significantly increase the risk of systemic infection (sepsis) because the clobetasol-treated area will be small (less than 5 % of body surface area). However, the data safety monitoring board will also monitor for this possibility by comparing trial patients with historic, SCORETEN-matched control patients to assess for any increase in the number of serious adverse events. As outlined in the recruitment section, at least three of the first five patients will need to survive in order for enrollment to proceed.

### Sample size and statistical considerations

A total of 15 participants will be needed to complete this study. The difference in days to cessation of skin detachment between clobetasol and placebo-treated skin will serve as the primary endpoint for the sample size calculation of the study. Let *μ* be the expected difference in number of days between the control and treatment groups, respectively. We wish to test the null hypothesis *μ* = 0 versus the alternative hypothesis *μ* ≠ 0. From our own clinical experience, we make an estimate, 4.1, for the population standard deviation for the difference in number of days to cessation of skin detachment between designated treatment areas on the right and left sides of the body. A sample size of 13 patients (26 contralateral treatment areas) is required to test the null hypothesis *μ* = 0 versus the alternative hypothesis |*μ*| = 3.5. For this calculation, we assumed the difference in number of days to cessation of skin detachment to be normally distributed and we used the two-sided paired *t* test with a power of 80 % at a significance level of 0.05. We will enroll 15 patients, to account for an estimated 15 % drop out rate.

### Statistical analysis of primary and secondary endpoints

Summary statistics will be generated for the time to skin detachment of the clobetasol and placebo-treated areas based on daily skin examinations. In addition, the secondary outcome measures (time to 90 % re-epithelialization, time to cessation of skin detachment, percentage affected surface area, and percentage surface area of detached skin) will be evaluated at 0, 3, 6, 9, 12, and 15 days. If the difference between treatment and control areas is normally distributed, then the two-sided paired *t* test will be used to assess whether there is a statistically significant difference in these outcome measures, that is, whether their difference is statistically significantly different from zero. The Shapiro–Wilk test will be used to assess for the normality of the difference in the outcome measures between treatment and control and a *Q*-*Q* plot will be used to verify test results. If the *P* value of the Shapiro–Wilk test is small (for example, < 0.05), we will use the two-sided Wilcoxon signed-rank test, instead of the paired *t* test, to assess whether there is a statistically significant difference in the measured outcome ranks between treatment and control, that is, whether the median difference in their paired values is statistically significantly different from zero. Since multiple endpoint measures will be compared, statistical correction for multiple testing is warranted. Tukey’s multiple comparison procedure will be applied to maintain the family-wise error rate at 0.05 for multiple comparisons.

## Discussion

Topical steroids are a first-line treatment for multiple cutaneous inflammatory diseases. In general, topical steroids are most effective when used to treat superficial inflammation involving the epidermis, upper dermis, or dermal-epidermal junction. Topical steroid application might in fact be superior to systemic corticosteroids for treating such conditions.

Bullous pemphigoid is an example of a superficial inflammatory dermatosis in which the immune system attacks proteins at the dermal-epidermal junction, resulting in widespread bullae and erosions. Topical steroids are the first-line treatment for bullous pemphigoid, and have been found to have superior efficacy and fewer systemic side effects when compared with oral corticosteroid therapy [37, 40, 41]. Cutaneous lupus erythematosus is another disease characterized by the presence of an inflammatory infiltrate and deposition of autoantibodies at the dermal-epidermal junction, and is also treated primarily with topical steroids [42]. Pyoderma gangrenosum is a rare neutrophilic dermatosis of uncertain etiology that commonly presents with inflammatory ulcers of the skin, and the cutaneous lesions have been shown to respond favorably to topical or intralesional steroids [43, 44]. To date, there are no published reports investigating the use of topical steroids such as clobetasol for treatment of the cutaneous manifestations of SJS or TEN.

Perhaps the most compelling reason to conduct the proposed study is that multiple studies in the ophthalmology literature support the use of topical steroids for the ocular manifestations of TEN [16, 45, 46]. The ocular sequelae of SJS and TEN occur as a result of the same pathologic process as the cutaneous aspect of the disease, with desquamation of the ocular surface resulting in pseudomembranous or membranous conjunctivitis [45] Additional ocular sequelae include adhesions between the bulbar and palpebral conjunctivae (symblepharon), as well as destruction of the corneal limbal stem cells leading to vascularization and thickening of the corneal epithelium [46, 47]. This ‘conjunctivalization’ of the cornea can lead to corneal opacification and severe visual loss [48]. Direct ocular application of high-dose topical steroids has been shown to decrease disease duration and improve visual outcomes in patients with TEN [16]. Sotozano and colleagues [16] compared the visual outcomes of 64 TEN patients treated with (33 patients) and without (31 patients) topical ocular steroids during the acute phase of the disease. Over 74 % of patients treated with topical steroids had 20/200 vision or better compared with just 21 % of the patients who were not treated with steroids. The percentage of patients with 20/20 vision or better was also increased in the topical steroid group (41 %) versus the untreated group (21 %). Importantly, patients who were not treated with steroids were more likely to have worse than 20/2,000 vision (41 % versus 21 %) and this was statistically significant (*P* <0.00001) [16]. Given that corneal and skin keratinocytes are affected by the same pathologic process in TEN, topical steroids might also be beneficial for the cutaneous manifestations of the disease.

The major evidence against treating TEN with steroids is a clinical study (*N* = 30) in which patients who were managed without corticosteroids had improved survival, a finding that trended towards but did not reach significance [30]. One criticism of this study is that 11 of 15 patients in the ‘steroid-free’ arm had actually received corticosteroids at external medical centers prior to enrollment. Thus, an alternative conclusion could be that steroids when given early might be beneficial but that prolonged steroid therapy might be detrimental. However, as further research has been done, it appears that the duration of steroid therapy might be an important factor, and several larger studies have shown a possible benefit. The EuroSCAR study compared supportive care, systemic corticosteroids, and systemic corticosteroids plus intravenous immunoglobulin therapy in 281 patients and found no significant survival benefit with either steroids or intravenous immunoglobulin therapy, although the benefit from corticosteroids trended towards significance [20]. A recent Spanish retrospective study of 12 children with TEN did not show any increase in mortality with systemic corticosteroid use [49], and a systematic literature review of pediatric patients with TEN also failed to show any increased mortality in the group treated with systemic steroids [50].

As with oral corticosteroids, topically applied steroids might lead to systemic adverse effects, such as suppression of the hypothalamic-pituitary axis, diabetes, and iatrogenic Cushing’s syndrome. Several studies have shown the systemic absorption and adverse effects of topical steroids to be higher when applied to mucous membranes (such as the oral cavity) and non-intact skin. A recent review described five cases of Cushing syndrome following topical use of steroids for inflammatory disorders of the oral mucosa [51]. A prospective study of adrenal suppressive effects in 43 patients treated with topical calcipotriene plus betamethasone diproprionate revealed that 4.5 % of patients developed signs of adrenal suppression (based on results of adrenocorticotropic hormone stimulation) after four weeks of therapy that were reversed following cessation of the medication [52]. In addition to hypothalamic-pituitary axis suppression, an increased risk of local or systemic infection is also a potential concern with topical steroid use. A recent case review describing infection risk in patients with bullous pemphigoid treated with topical clobetasol found that 9 of 30 patients developed a cutaneous infection ranging from cellulitis in 6 patients to fatal necrotizing fasciitis in 3 patients [53]. The presence of other comorbidities that could influence infection risk was also noted, including diabetes and immunosuppression. Patients who developed infectious complications had a higher prevalence of diabetes (33 % versus 19 %) than patients who did not develop cutaneous infections. In addition, two of the three patients who developed necrotizing fasciitis were also receiving systemic immunosuppressive medications.

Several features of this study aid in minimizing the potential risks of topical steroid treatment and aid in early identification of potential complications. First, the area of application will be small and limited to 10 % of the total body surface area between the control and treatment areas. Systemic absorption of topical medications is related to both skin integrity as well as body surface area. Given the extensive damage to the skin architecture and loss of barrier function in TEN, we expect that any topically applied medications are going to have a much higher rate of absorption compared with application on intact skin. This is true in many immunocutaneous diseases that are treated with topical steroids, including pemphigus vulgaris and erosive discoid lupus erythematosus. Thus, by limiting the amount of body surface area treated, we hope to minimize both potential local and systemic adverse effects of the topical steroids. Secondly, the duration of treatment will be short and daily systematic evaluations will identify discrepancies in the skin appearance that could be indicative of worsening disease or local infection. Though the control arm might experience some effect from systemically absorbed steroids, the concentration delivered to the skin through systemic absorption would be comparatively lower than the arm treated topically.

Several potential limitations of the study design merit mention. As the split-body design designates patients as their own controls, accurate outcome comparison between the steroid and placebo-treated skin will depend partially on the selection of appropriately matched treatment areas. While treatment areas on opposite sides of the body will be selected on the basis of similar cutaneous involvement by TEN, the areas might be at different stages of progression (that is, if lesions appeared at different times). Thus, an older lesion might re-epithelialize faster than a comparable-appearing lesion that started more recently, independently of any topical treatments that are applied, and this might confound the results of treatment. To minimize this, investigators will try to ascertain the pattern of disease progression and attempt to select sites with similar levels of involvement as well as comparable times of onset. However, in some cases, this information may not be available from the patient history, especially for patients who have been transferred from other medical centers or are unable to provide an accurate clinical history. Secondly, the time to complete re-epithelialization in TEN can be variable, ranging from 7 to 20 days in several case series evaluating various therapies for the disease [54–57]. The time to 90 % re-epithelialization is a secondary endpoint measure of the study; based on reported times to complete re-epithelialization, the 15-day follow-up period is felt to be reasonable time to evaluate this outcome measure. However, there is a possibility that the selected timeline will be too short; if the majority (>50 %) of patients do not reaching the secondary endpoint within the 15-day study timeline, the follow-up period might need to be increased. Finally, vasoconstriction is a known pharmacologic property of topical steroid preparations and this effect could potentially compromise the blinded design of the study [58]. While vasoconstriction is a property of topical steroid preparations, to date the clinical studies of vasoconstriction have been performed on healthy, intact skin. In the setting of TEN with extensive erythema, inflammation, and desquamation of the skin, it might be difficult to predict the extent of the blanching effect from the topical steroid application and whether it could be a confounding factor in the blinding of the study. Furthermore, several randomized, blinded split-body trials comparing steroids with emollients or other non-steroidal topical preparations have been successfully performed and published in the dermatologic literature, namely comparing topical steroids with emollients for treatment of atopic dermatitis; in these studies the vasoconstrictive properties of topical steroid preparations was not significant enough to interfere with blinding [59–64].

The proposed study would be the first randomized controlled trial to evaluate the safety and efficacy of topical steroids for the cutaneous manifestations of TEN. Topical steroids have shown promising results in reducing ocular disease duration, severity, and long-term visual sequelae in patients with TEN. As the skin and ocular manifestations in TEN are occurring as a result of the same pathologic process, topical steroids might also be beneficial for the cutaneous aspects of the disease. By performing topical steroid application in a well-controlled and limited manner, we hope to characterize its effect on disease progression as well as any potential adverse effects. We hypothesize that clobetasol treatment will promote epidermal keratinocyte survival through suppression of TNF signaling and inhibition of apoptosis, resulting in shorter disease duration and decreased time to re-epithelialization. In addition, we predict that the genomic analysis of skin biopsy specimens before and after steroid treatment will demonstrate differential expression of pro-apoptotic genes. This trial will serve as a proof-of-concept study to assess safety and efficacy, and may serve as a basis for future large controlled trials of topical steroids or other immunosuppressive agents in the treatment of SJS and TEN.

## Trial status

This protocol is for a proposed clinical trial. The phase I protocol has been reviewed and approved by the University of California Davis Institutional Review Board in December 2014. This trial has been registered through clinicaltrials.gov on 8 December 2014 and is accessible online (NCT 02319616). Patient recruitment has not yet commenced for this trial.

## Abbreviations

FDA: US Food and Drug Administration
miRNA: microRNA
PCR: polymerase chain reaction
SCORETEN: SCORe of Toxic Epidermal Necrosis
SJS: Stevens–Johnson syndrome
SNP: single nucleotide polymorphism
TEN: toxic epidermal necrolysis
TNF: tumor necrosis factor
